# A208 MENTAL HEALTH, WORK IMPAIRMENT, AND MEDICATION SATISFACTION AMONGST INDIVIDUALS WITH INFLAMMATORY BOWEL DISEASE RECEIVING LONG-TERM BIOLOGIC AND ADVANCED TREATMENT

**DOI:** 10.1093/jcag/gwae059.208

**Published:** 2025-02-10

**Authors:** E Hazan, E Stefanova, A Afshar, A Patel, L Targownik

**Affiliations:** Internal Medicine, University of Toronto, Toronto, ON, Canada; Mount Sinai Hospital, Toronto, ON, Canada; Mount Sinai Hospital, Toronto, ON, Canada; Mount Sinai Hospital, Toronto, ON, Canada; Internal Medicine, University of Toronto, Toronto, ON, Canada

## Abstract

**Background:**

Biologic and advanced immunotherapies have been shown to be effective in controlling biochemical, endoscopic, and histologic disease activity in individuals with Inflammatory Bowel Disease (IBD). However, there is limited data assessing the prevalence of somatic symptoms, mental health, and quality of life (QoL) in this population. To provide holistic care and appropriately manage both gut and somatic symptoms, it is critical to assess the prevalence of these symptoms and impairments.

**Aims:**

This study assessed the prevalence of mental health deficits, quality of life, and work impairment in IBD patients who are being treated with biologic and advanced therapies for at least six months. The objective was to assess whether significant gaps in these domains persist as well as whether individuals are satisfied with their current advanced treatment.

**Methods:**

Cross-sectional data was prospectively collected using a survey completed by individuals >18 years old, with a known diagnosis of Ulcerative Colitis, Crohn’s Disease, or Indeterminate Colitis, who have been receiving a biologic or advanced immunotherapy at a stable dose for >= 6 months. In addition to demographic data, the survey contained validated questionnaires assessing mental health, work impairment, and QoL. The presence of deficits in these domains was then evaluated using absolute scores and validated cutoffs.

**Results:**

This study included 37 individuals who completed the survey as well as an additional 3 who completed part of it. 85% of respondents agreed that their current therapy has improved their symptoms and 62.5% of respondents are satisfied with their current treatment. 5.4-29.7% and 13.5-29.7% endorsed symptoms consistent with depression and anxiety respectively on different questionnaires. 51.4% of respondents endorsed active symptoms, and 56.8% endorsed symptom severity consistent with non-remission. A mean of 2.74 hours of weekly work productivity were lost due to IBD activity.

**Conclusions:**

The majority of individuals with IBD on long-term advanced therapy report symptom improvement and satisfaction with their treatment. However, more than half endorsed ongoing symptoms of IBD, including work impairment, and nearly one-third endorsed mental health impairment consistent with depression or anxiety. This suggests that despite perceived treatment efficacy, significant gaps in these domains persist.

**Funding Agencies:**

None

